# Uncovering Actions of Type 3 Deiodinase in the Metabolic Dysfunction-Associated Fatty Liver Disease (MAFLD)

**DOI:** 10.3390/cells12071022

**Published:** 2023-03-27

**Authors:** Rafael Aguiar Marschner, Ana Cristina Roginski, Rafael Teixeira Ribeiro, Larisse Longo, Mário Reis Álvares-da-Silva, Simone Magagnin Wajner

**Affiliations:** 1 Endocrine Division, Hospital de Clínicas de Porto Alegre, Universidade Federal do Rio Grande do Sul, Porto Alegre 90035-003, RS, Brazil; 2 Post-Graduate Program in Biochemestry, Universidade Federal do Rio Grande do Sul (UFRGS), Porto Alegre 90035-003, RS, Brazil; 3 Graduate Program in Gastroenterology and Hepatology, Universidade Federal do Rio Grande do Sul, Porto Alegre 90035-003, RS, Brazil; 4 Experimental Laboratory of Hepatology and Gastroenterology, Center for Experimental Research, Hospital de Clínicas de Porto Alegre, Porto Alegre 90035-903, RS, Brazil; 5 Department of Internal Medicine, Universidade Federal do Rio Grande do Sul (UFRGS), Porto Alegre 90035-003, RS, Brazil

**Keywords:** thyroid metabolism, Krebs cycle, MAFLD, type 3 deiodinase

## Abstract

Metabolic dysfunction-associated fatty liver disease (MAFLD) has gained worldwide attention as a public health problem. Nonetheless, lack of enough mechanistic knowledge restrains effective treatments. It is known that thyroid hormone triiodothyronine (T3) regulates hepatic lipid metabolism, and mitochondrial function. Liver dysfunction of type 3 deiodinase (D3) contributes to MAFLD, but its role is not fully understood. Objective: To evaluate the role of D3 in the progression of MAFLD in an animal model. Methodology: Male/adult Sprague Dawley rats (n = 20) were allocated to a control group (2.93 kcal/g) and high-fat diet group (4.3 kcal/g). Euthanasia took place on the 28th week. D3 activity and expression, Uncoupling Protein 2 (UCP2) and type 1 deiodinase (D1) expression, oxidative stress status, mitochondrial, Krebs cycle and endoplasmic reticulum homeostasis in liver tissue were measured. Results: We observed an increase in D3 activity/expression (*p* < 0.001) related to increased thiobarbituric acid reactive substances (TBARS) and carbonyls and diminished reduced glutathione (GSH) in the MAFLD group (*p* < 0.05). There was a D3-dependent decrease in UCP2 expression (*p* = 0.01), mitochondrial capacity, respiratory activity with increased endoplasmic reticulum stress in the MAFLD group (*p* < 0.001). Surprisingly, in an environment with lower T3 levels due to high D3 activity, we observed an augmented alpha-ketoglutarate dehydrogenase (KGDH) and glutamate dehydrogenase (GDH) enzymes activity (*p* < 0.05). Conclusion: Induced D3, triggered by changes in the REDOX state, decreases T3 availability and hepatic mitochondrial capacity. The Krebs cycle enzymes were altered as well as endoplasmic reticulum stress. Taken together, these results shed new light on the role of D3 metabolism in MAFLD.

## 1. Introduction

Metabolic dysfunction associated fatty liver disease (MAFLD) is the most common form and the leading cause of morbidity and mortality among liver diseases [1]. The prevalence of MAFLD is around 25–38% of the world population [2,3]. Although the risk factors associated with the disease and its progression are well described in the literature, the pathophysiological mechanisms involved are still unclear [4]. The early stages MAFLD are characterized by the accumulation of fat in the liver and chronic inflammation [5]. Other mechanisms less studied include T3 regulation, which affects signaling pathways, gene induction, and protein expression [6].

Thyroid hormones (THs) are essential for growth, development, and metabolism. While thyroxine (T4) is the main product of the thyroid, the biologically active hormone is triiodothyronine (T3). Both THs enter cells via specific transporters and are regulated locally, inside each tissue, by the activity of types 1 (D1), 2 (D2), and 3 (D3) deiodinases. Deiodinases types D1 and D2 convert intracellular T4 to T3 [7], while D3 converts T3 to rT3 and other inactive forms of the hormone [8]. Type 3 deiodinase is known to be augmented in several types of diseases, leading to altered local T3 amounts in several tissues, such as the heart and muscle [9,10]. In the liver, THs directly influence the metabolism of lipids and carbohydrates, through the processes of hepatic lipogenesis, lipid oxidation, cholesterol homeostasis, and gluconeogenesis [11,12,13]. It was recently verified that T3 levels in the hepatic tissue are reduced in MAFLD [14] resulting in a reduction in lipolysis and, consequently, impairment of triglyceride metabolism, and β-oxidation of free fatty acids (FFAs). On the other hand, once T3 increases its availability in tissue, the compromised parameters improve, reorganizing the hepatic network, mitochondrial turnover, and hepatic autophagy [15,16].

Type 3 deiodinase is known to directly alter T3 availability through augmenting T4 and T3 inactivation under inflammation and altered REDOX status, thereby modifying mitochondrial function, the Krebs cycle, and endoplasmic reticulum stress. Interestingly, there are few studies on the mechanisms involving the activation of D3 in liver tissue and its dysfunctions in MAFLD. Thus, this study aimed to evaluate how D3 dysfunction may be associated with disease in the liver of an animal model of MAFLD.

## 2. Materials and Methods

### 2.1. Animals and Procedures

Male Sprague Dawley rats (weighing between 250–350 g) were used in the experiments. All procedures and experiments involving animals followed the recommendations of the Brazilian College of Animal Experimentation (COBEA). Our study followed the ethical principles of the Guide for the Care and Use of Laboratory Animals [17] and the Guidelines for Reporting Animal Research international standards for animal research [18]. The Research Ethics Committee of our institution and the Animal Ethics Committee of the Hospital de Clínicas de Porto Alegre approved this study (Protocol nº 2019-0297). Briefly, all animals received standard chow or high-fat choline-deficient diet (HFCD) (Rhoster Ltda, Araçoiaba da Serra, Brazil) and water ad libitum throughout the experiment. They were maintained in a 12 h sleep/wake cycle, at a temperature of 22 ± 1 °C and with air exhaustion.

The experimental model was carried out by previous studies [19]. A total of 20 rats were allocated into two groups: the control group (n = 10) received a standard diet of 2.93 kcal/g for 28 weeks and the MAFLD group (n = 10) received a choline-deficient hyper lipidic diet of 4.3 kcal/g (31.5% lipids, enriched with 54.0% trans fatty acids) for 28 weeks [19]. At the end of the experimental period, the animals were sacrificed (anesthetized with a vaporizer (Surgivet, Saint Louis, MN, USA)) and the liver was removed, frozen in liquid nitrogen, and stored at −80 °C.

### 2.2. Biochemical Parameters

Serum analyses of triglycerides, total cholesterol, high-density lipoprotein (HDL), low-density lipoprotein (LDL), and glucose was performed.

### 2.3. Hepatic Histology and Immunofluorescence

Liver tissue samples were fixed in formalin and embedded in paraffin, later stained with hematoxylin and eosin (H&E) and picrosirius red, performed according to Liang et al. [20]. Assessment of the degree of steatosis was performed by a pathologist blinded to the experimental groups. Fibrosis was quantified by morphometric analysis from picrosirius staining. Ten photos of randomly selected fields were obtained per animal, using the Olympus BX51 microscope, QCapture X64 program with 200× magnification to determine the labeling intensity. This evaluation was performed using the ImageJ program (version 1.51p, National Institutes of Health, Bethesda, MA, USA).

The immunofluorescence technique was performed according to Solari et al. [21], with some modifications. Briefly, liver samples fixed in 10% buffered formalin and embedded in paraffin were microtome cut at 3 µm, placed on a silanized slide, and deparaffinized. Protein blocking was performed with 3% BSA for 1 h at room temperature. Slides were permeabilized with 0.05% Tween 20 diluted in PBS. The incubation with the primary antibodies was overnight at 4 °C, the dilutions used were D3 (NBP1-05767, novusbio, Englewood, CO, USA) 1:400. Incubation with secondary antibody was performed for 1 h 30 min at room temperature with anti-rabbit IgG secondary antibody (A11008, Invitrogen-Thermo Fischer, Waltham, MA, USA), at 1:1000 dilution. The blades were mounted with Fluoroshield mounting medium with Dapi (Abcam, ab104139, Cambridge, UK).

### 2.4. Inflammatory Markers

The detection of inflammatory markers was performed using the ELISA technique. Serum concentrations of IL-6 and TNF-α (Invitrogen, Waltham, MA, USA) were evaluated according to the manufacturer’s instructions. All analyses were performed in duplicate. The absorbance was measured in a spectrophotometer at a wavelength of 450 nm (Zenyth 200rt, New York, NY, USA). Results were expressed in pg/mL.

### 2.5. Oxidative Stress Parameters

#### 2.5.1. Carbonyl Content

Carbonyl content was measured according to Zannata et al. (2013) [22]. The difference between the samples treated with 2,4-dinitrophenylhydrazine and treated with HCl (white) was used to calculate the carbonyl content determined at 370 nm. The data obtained was calculated by the millimolar absorption coefficient of hydrazine (e370 nm = 21.000000·M^−1^·cm^−1^), and the results were expressed in nmol carbonyl/mg of protein.

#### 2.5.2. Malondialdehyde Levels

The technique to assess MDA concentrations was based on the method of Yagi [23]. The fluorescence of the organic phase was read at wavelengths of 515 and 553 nm of excitation and emission, respectively. A calibration curve was performed with 1,1,3,3-tetramethoxypropane and subjected to the same treatment as the supernatant. MDA levels were calculated as nanomoles of MDA/mg protein.

#### 2.5.3. Sulfhydryl Content

The sulfhydryl content was determined, as described by Aksenov and Markesbery [24], where 5-thio-2-nitrobenzoic acid (TNB) derived from the reaction of thiols with 5,5′-dithiobis (2-nitrobenzoic acid) forms a yellow-colored derivative that is read in a spectrophotometer, measuring the absorbance at 412 nm. Results were expressed as nmol TNB/mg protein.

### 2.6. Antioxidant Defenses

#### 2.6.1. Reduced Glutathione Concentrations

The GSH parameter was measured, as described by Browne and Armstrong [25]. Fluorescence was measured using excitation and emission wavelengths of 350 and 420 nm, respectively. The calibration curve was prepared with standard GSH (0.001–1 mM) and concentrations were calculated as nanomoles of GSH/mg protein.

#### 2.6.2. Glutathione Peroxide Activities

The glutathione peroxidase (GPx) assay was performed according to Wendel [26]. Enzyme activity was determined by monitoring the disappearance of NADPH at 340 nm. The unit of GPx (U) was defined as 1 μmol of NADPH consumed per minute. Specific activity was calculated as U/mg of protein.

#### 2.6.3. Glutathione Reductase Activities

The glutathione reductase (GR) activity was performed according to Carlberg and Mannervik [27]. Enzyme activity was determined by monitoring NADPH consumption at 340 nm. One GR U is defined as 1 μmol of GSSG reduced per minute. Specific activity was calculated as U/mg of protein.

#### 2.6.4. Superoxide Dismutase Activities

Superoxide dismutase (SOD) activity was performed according to the study by Marklund [28]. The absorbance was read at 420 nm. A calibration curve with purified SOD as a standard was used to calculate the SOD activity present in the samples. Specific activity was calculated as U/mg of protein.

### 2.7. Real-Time PCR

Total RNA was extracted from tissues by the trizol method, cDNA was synthesized (SuperScript First-Strand Synthesis System for RT-PCR; Invitrogen), followed by real-time PCR with SYBR Green PCR Master Mix (Table 1, Applied Biosystems, Waltham, MA, USA) in ABI Prism 7500 Sequence Detection System Assay (Applied Biosystems). The r2 was greater than 0.99 and the amplification efficiency varied between 80% and 100%. Samples were measured by relative quantification (change in expression in MAFLD vs. Control group).

### 2.8. Mitochondrial Capacity

#### 2.8.1. Mitochondrial Respiratory Parameters (Oxygen Consumption)

The rate of oxygen consumption was measured using an OROBOROS Oxygraph-2k (Innsbruck, Austria) in a thermostatically controlled (37 °C) and magnetically stirred incubation chamber [29], with modifications [30]. The assay was performed with crude liver homogenates (1 mg tissue· mL^−1^) and incubated in MIR 05 buffer containing 0.5 mM EGTA, 3 mM MgCl_2_, 60 mM K-lactobionate, 20 mM taurine, 10 mM KH_2_PO_4_, 20 mM HEPES, 110 mM sucrose, 1 g/L BSA, pH 7.1.

Oxygen consumption was measured through the substrate-uncoupler inhibitor titration (SUIT) protocol [29]. Oxidative phosphorylation (OXPHOS) capacity (state 3 respiration) was determined by NADH-linked substrates (5 mM pyruvate, 0.5 mM malate, and 10 mM glutamate (PMG) followed by 1 mM ADP to determine the “State 3—PMG”. Following the protocol, the “State 3—PMG+S” was obtained by supplementation of 10 mM succinate (FADH 2 -linked substrate). Oligomycin (1 μg mL^−1^) was used to obtain the resting respiration “State 4”. Next, 1.5 µM CCCP (three pulses of 0.5 µM) was supplemented to induce the noncoupled respiration “Noncoupled respiration PMG+S”, and 2 µM rotenone (complex I inhibitor) was used to obtain the noncoupled respiration stimulated by succinate “Noncoupled respiration S”. Finally, 2.5 µM antimycin A (complex III inhibitor) was used to inhibit the transfer of electrons from heme bH to oxidized Q, and to block all remaining mitochondrial respiration. The real-time oxygen fluxes were calculated using DatLab7 (Oroboros Instruments, Innsbruck, Austria) and expressed as pmol O_2_ flux·s ^−1^ ·mg protein ^−1^.

#### 2.8.2. Complexes II, II-III, and IV

The liver was homogenized (1:10 w/v) in SETH buffer pH 7.4, centrifuged at 800× *g* for 10 min at 4 °C and the supernatant was aliquoted and subjected to three cycles of freezing and thawing to rupture the mitochondrial membranes. Afterward, it was used to determine the activity of the electron transport chain complexes: Complex II–III, analyzed through the reduction of cytochrome C at 550 nm [31]; Complex IV, evaluated through the oxidation of cytochrome C at 550 nm [32]; and Complex II, evaluated by the determination of succinate-2,6-dichloroindo-phenol (DCIP)-oxidoreductase activity in 600 nm [32]. The absorbance variation was detected by a Spectramax M5 microplate spectrofluorometer and the activities of the complexes were calculated, and data were expressed as nmol·min^−1^·mg protein^−1^.

#### 2.8.3. Activities of Glutamate Dehydrogenase (GDH), α-Ketoglutarate Dehydrogenase (α-KGDH) and Succinate Dehydrogenase (SDH)

The activities of GDH [33] and α-KGDH [34] were determined by a Spectramax M5 microplate spectrofluorometer using crude liver homogenate (GDH: 0.15 mg protein mL^−1^; α-KGDH: 0.25 mg protein mL^−1^). The activity of both enzymes was determined through the reduction in NAD^+^ and expressed as nmol·min^−1^·mg protein^−1^. The SDH activity [32] was measured using liver homogenate (0.15 mg protein mL^−1^), and the reduction in 2,6-dichloroindophenol (DCIP) at 600 nm, data expressed as nmol·min^−1^·mg protein^−1^.

### 2.9. Western Blot Analyses

Liver samples were prepared as described [35]. Briefly, 30–50 μg of protein from each sample was fractionated by 8–12% SDS-PAGE and transferred to an Immobilon PVDF membrane (Millipore, Billerica, MA, USA). The following primary antibodies were used: anti-D3 (1:1000; Novus Biologicals, Englewood, CO, USA); anti-ERK1 (1:500; Santa Cruz Biotechnology, Dallas, TX, USA); anti-MAPK (1:1000; Cell Signaling Technology, Danvers, MA, USA); anti-p38 (1:1000; Cell Signaling Technology); anti-GRP78 (1:700; Abcam) and anti-β-actin (1:25,000; Sigma-Aldrich, St. Louis, MA, USA). Antigen-antibody complexes were visualized using HRP-conjugated secondary antibody and an enhanced chemiluminescence system (GE Healthcare, Pittsburgh, PA, USA). Expression was quantified using image densitometry with ImageJ analysis software.

### 2.10. D3 Activity

The D3 activity of liver samples was determined using the paper chromatography technique as described by Huang et al. (2005) [36]. Briefly, tissues were homogenized and sonicated with 10 mM Tris-HCl, 0.25 sucrose buffer (pH 7.5), and 10 mM dithiothreitol (DTT). The amount of protein was measured by the Bradford assay. Homogenized livers were incubated for one hour with 200,000 cpm of ^125^I-labeled T3, 2 nM T3, 20 mM DTT, and 1 mM propylthiouracil (PTU) to inhibit any D1 activity. The addition of 200 nM of T3 completely abolished D3 activity in all samples. The reaction was stopped with 200 μL of 95% ethanol, 50 μL of NaOH (0.04 N) and 5 mg of PTU. Deiodination was determined based on the amount of 125 I-3,3′-T2 generated. Results were expressed as the fraction of T2 counts minus nonspecific deiodination (always <1.5%), obtained with the saturating concentration of T3 (200 nM). D3 activity data were expressed as T3 fentomoles per minute per milligram of protein. 

### 2.11. Statistical Analysis

Unless otherwise specified, results are presented as mean ± SD. Data were analyzed using a 2-tailed Student’s *t*-test or 1-way ANOVA followed by post hoc Duncan multiple-range tests when F was significant. Prism 9.0 software (Graphpad, San Diego, CA, USA) was used for statistical analysis; a *p* value less than 0.05 was considered significant.

## 3. Results

### 3.1. Sample Characteristics and Biochemical Parameters 

The body weight of animals in the MAFLD group was significantly higher compared to their control (*p* < 0.01). All biochemical parameters were altered in the MAFLD group (Table 2). 

### 3.2. MAFLD Generates Histopathological Changes

**Figure 1:**
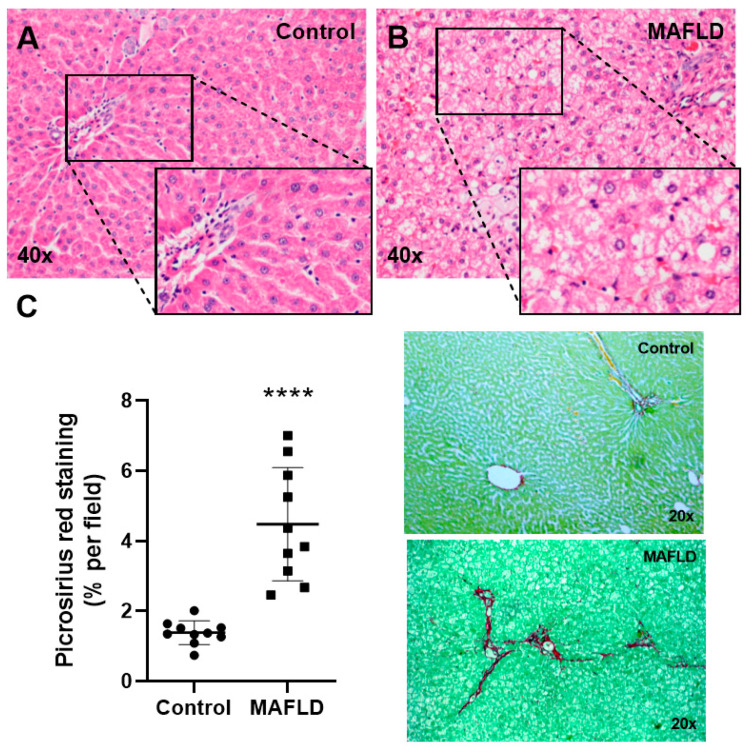
Histological evaluation of the liver. Control (**A**) and MAFLD (**B**) groups, H&E staining, at 40× magnification. Assessment by Picrosirius (**C**), the MAFLD group showed an increase for fibers. **** *p* < 0.01 vs. Control, n = 10 per group.

Animals in the control group did not show abnormalities in the liver (Figure 1A). Nevertheless, animals in the MAFLD group presented moderate intensity of microvesicular and macrovesicular steatosis, in addition to a mild degree of fibrosis (Figure 1B). Collagen quantification demonstrated a higher amount of connective tissue fibers (*p* < 0.01) in the MAFLD group compared to the control group (Figure 1C).

### 3.3. MAFLD Induces Inflammatory and REDOX State Parameters 

**Figure 2:**
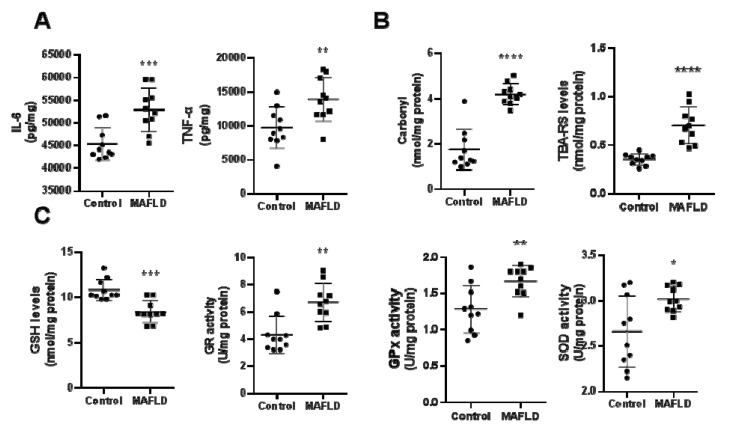
Evaluation of inflammatory markers, IL-6 and TNF-α, in the liver (Panel (**A**)). The quantification of IL-6 and TNF-α were shown to be increased in the MAFLD group. As for the REDOX state, we evaluated the content of carbonyl groups and TBA (Panel (**B**)) and levels of GSH, GPx, GR and SOD activity (Panel (**C**)) in the liver. Carbonyl and TBA were increased in the MAFLD group (**B**). GSH content was decreased in liver samples from MAFLD animals (**C**), while GPx, GR and SOD activity were increased in the MAFLD group (**C**). * *p* < 0.05; ** *p* < 0.01; *** *p* < 0.001; **** *p* < 0.0001 vs. Control, n = 10 in each group.

The hepatic concentrations of the pro-inflammatory cytokines IL-6 (*p* < 0.05) and TNF-α (*p* < 0.01) were higher in the MAFLD group compared to the control group (Figure 2A). Protein and lipid damage in liver tissue were assessed with carbonyl and MDA assays, respectively. Carbonyl and TBARS (*p* < 0.001) were increased in the MAFLD group (Figure 2B). We observed reduced GSH levels (*p* < 0.01) and increased activities of GPx, GR and SOD enzymes in MAFLD animals (Figure 2C). These results suggest an increase in reactive oxygen species induced by HFCD. 

### 3.4. Altered Mechanisms of Thyroid Hormone Metabolism in MAFLD 

**Figure 3:**
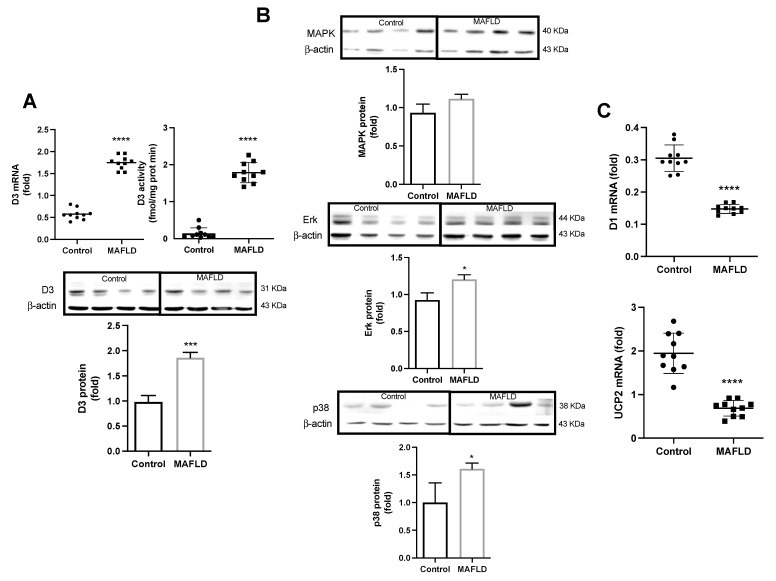
Assessment of thyroid metabolism in the liver (Panel **A**). D3 mRNA expression, activity and protein quantification increased in MAFLD animals (**A**). Evaluation of the mechanisms involved with D3 (Panel **B**). The MAPK pathway showed an increase of 11% in protein quantification, while its subsequent ERK and p38 pathways showed an increase of 21 and 68%, respectively in MAFLD animals. Expression of genes linked or stimulated by T3 (Panel **C**). D1 and UCP2 mRNAs showed a reduction in MAFLD animals (**C**). * *p* < 0.03, *** *p* < 0.0005, **** *p* < 0.0001 vs. Control, n = 10 in each group.

**Figure 4:**
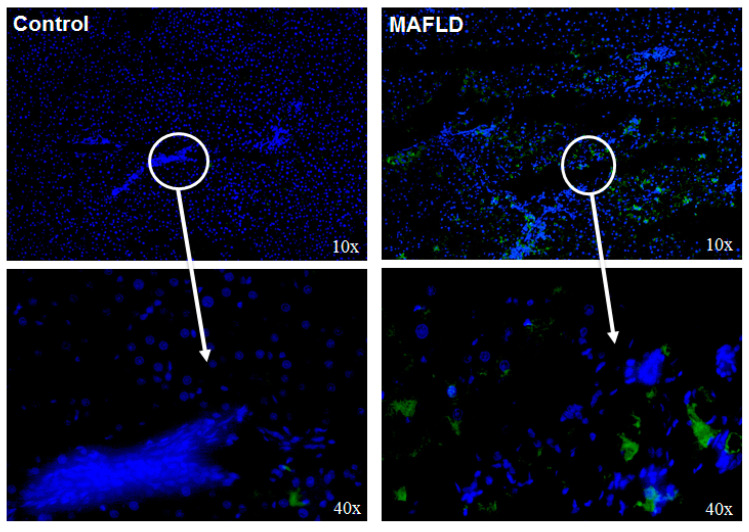
Assessment of thyroid metabolism in the liver. D3 immunofluorescence was increased in MAFLD animals.

We then determined the expression and activity of D3, the enzyme that inactivates T3 in disease. There was a significant increase in its expression, and activity (*p* < 0.0001) in MAFLD animals (Figure 3A). Also, an increased protein immunofluorescence staining was verified (Figure 4). We next sought to determine the pathways that increase D3 in the MAFLD liver. While the MAPK pathway augmented in 11%, ERK increased in 21%, and p38 increased in 68% (Figure 3B). There was a reduced expression of D1 and UCP2 mRNAs (*p* < 0.0001), genes positively stimulated by T3 hormone in the liver of the MAFLD group animals (Figure 3C). 

### 3.5. Mitochondrial Complexes Are Affected in MAFLD

**Figure 5:**
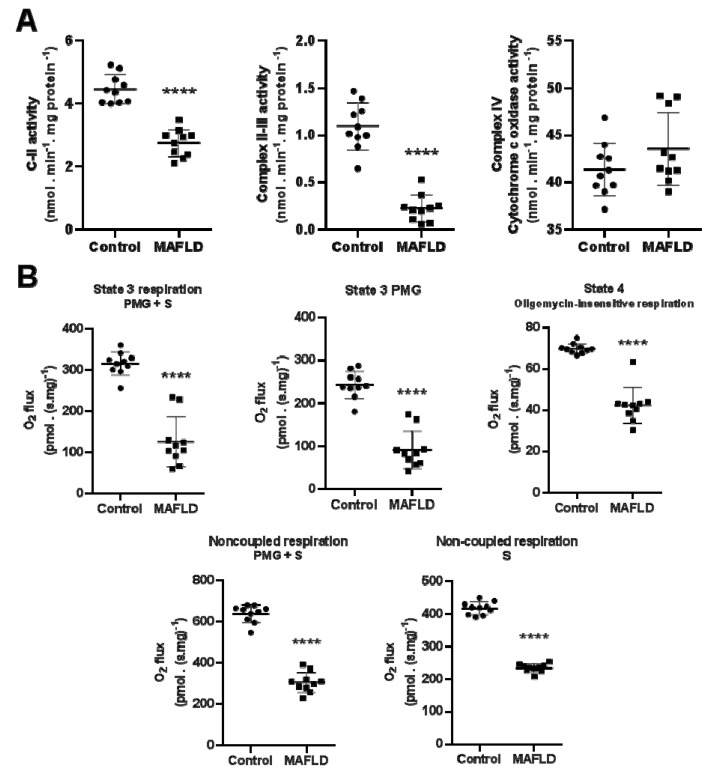
Assessment of mitochondrial respiratory complexes and capacity in the liver. The activity of complexes II and II-III were reduced in the MAFLD group, not observed in complex IV (**A**). The liver respiratory capacity was reduced in MAFLD animals, as state 3 (stimulated by PMG and PMG+S) and state 4 (stimulated by oligomycin) parameters were decreased (**B**). The same can be observed in the uncoupled states (stimulated by S and by PMG+S) (**B**). **** *p* < 0.0001 vs. Control, n = 10 in each group.

The complex II and II-III activity were reduced (*p* < 0.0001) in the MAFLD group (Figure 5A). No difference between groups was observed with complex IV activity (*p* = 0.0986, Figure 5A). 

### 3.6. MAFLD Induced Impairment of Mitochondrial Capacity

MAFLD animals showed reduced hepatic respiratory capacity. State 3 capacity (stimulated by PMG) had a significant decrease (*p* < 0.01) in MAFLD group. The same was observed in state 3 when stimulated by PMG+S and respiration in state 4 (stimulated by oligomycin, *p* < 0.01). Finally, the uncoupled states (stimulated by S and by PMG+S) also showed a significant reduction in their respiratory capacity (*p* < 0.0001, Figure 5B).

### 3.7. MAFLD Induces GDH, α-KGDH, SDH Enzymes and Endoplasmic Reticulum Stress

**Figure 6:**
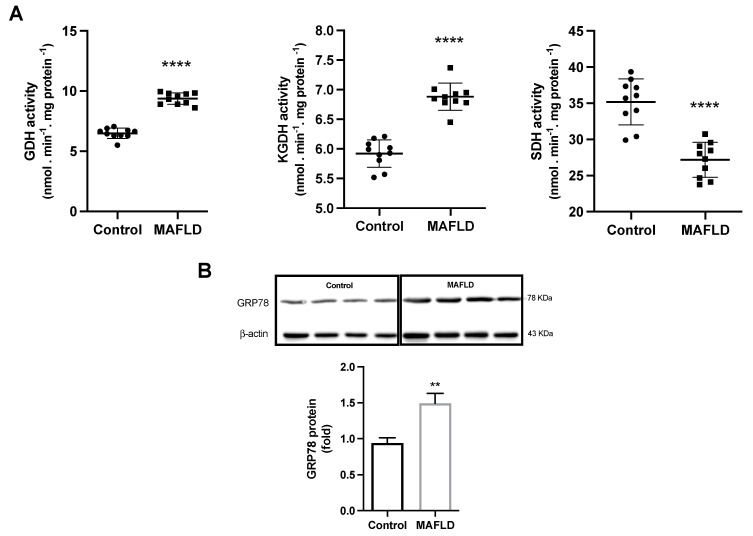
GDH, α-KGDH, SDH activity and GRP78 protein in the liver. The activity of the enzymes GDH and α-KGDH were increased while SDH activity decreases in MAFLD animals (**A**). GRP78 showed a 58% increase in the MAFLD group compared to the control group (**B**). ** *p* < 0.001 **** *p* < 0.0001 vs. Control, n = 10 in each group.

As the next step we evaluated the effect of HFCD on the enzymes that have a fundamental role in the Krebs cycle in the conversion of α-ketoglutarate to succinyl-CoA (α-KGDH) and succinate to fumarate (SDH). The activities of the enzymes GDH and α-KGDH were increased (*p* < 0.0001 both) while the activity of SDH was reduced (*p* < 0.0001) in the MAFLD (Figure 6A). Interestingly, these enzymes are not only correlated with disease progression, as expected, but also with type 3 deiodinase activity. Furthermore, when we evaluated the endoplasmic reticulum, we found that the MAFLD animals had a 58% increase in reticulum stress (Figure 6B). 

## 4. Discussion

The present study examines the effect of type 3 deiodinase on T3 levels in metabolic dysfunction-associated fatty liver disease (MAFLD). The results showed that the augmented IL-6, TBARS, and carbonyls increase, resulting in oxidative stress, and inducing D3 expression and activity. The decrease in GSH and sulfhydryl levels is parallel with the increased D3 function. In addition, GPx, GR, and SOD enzymes were altered, resulting in a pro-oxidative state. Importantly, UCP2 and Dio1 expressions were low, showing a decrease in local levels of T3. Moreover, while the mitochondrial capacity is diminished, an increase in the GDH and α-KGDH enzymes is observed. Together with the observed endoplasmic reticulum stress, this set of results adds to the current knowledge on the role of type 3 deiodinase and T3 on MAFLD.

The expression of D3 in MAFLD, so far under-explored, leads to a whole cascade of alterations. The study by Sinko et al. reported that TH levels alone are not the best marker of tissue THs signaling [37]. Nevertheless, the mechanisms involved in the activation of THs in the tissue become a good indicator of thyroid metabolism. Previous studies found that changes in the inflammatory profile and REDOX status increase D3 activity in acute illness situations [38,39,40]. Here, we observed that in a model of chronic disease, the accumulation of hepatic fat, via high-fat diets, induces inflammatory changes and a consequent increase in the production of reactive oxygen species (ROS) [14,41,42,43,44,45]. These data reinforce the direct association between the inflammatory profile and the REDOX state with diminished T3 levels, via an increase in D3 activity and a reduction in D1. The interaction between T3 and mitochondria is complex. T3 controls UCP2 and can act as a transporter for fatty acids, modulating mitochondrial metabolic activity. Moreover, the interaction involving T3 tightly controls Ca2+ in the mitochondria membrane and its metabolic activity [46,47]. T3 amount affects the turnover of the Krebs cycle, which in turn are strongly dependent on dehydrogenases in the mitochondrial matrix [48]. The altered D3 activity, which speeds T3 inactivation can be another factor that explains the lower energy production and the altered electron transport chain in the Krebs cycle, which reduces the respiratory capacity and causes mitochondrial dysfunction in MAFLD. 

The results shown here also clarify the data described in recent studies that used THR-β analogs [49,50]. The TG68 (THR-β agonist) used in an animal model of MAFLD increases the sensitivity of THR-β in the liver, increasing the availability of T3 to activate genes linked to the hepatic metabolism such as Acyl-CoA Oxidase-1 (ACOX1), carnitine palmitoyltransferase-1 (CPT-1), and D1. In addition, TG68 exerts an anti-steatogenic effect, improving 30% of fat liver content [49]. The increase in receptor availability improves liver function and increases the metabolism of lipids and lipoproteins [50]. Nevertheless, these agonists alone were able to ameliorate only a part of the whole disease process, suggesting that other adjuvant therapeutics are necessary to stabilize the disease.

In addition, we show that increased D3 activity in the liver occurs secondary to the augmented MAPK signaling pathway, via ERK and p38, which are already positively related to the expression and activity of D3 [51]. The increased expression in p38 and ERK in MAFLD can be due to increased hypoxia and cellular apoptosis, perpetuating a cycle of metabolic dysfunction in the liver and probably accelerating disease progression [52]. A former report revealed an inverse correlation between UCP2 mRNA expression and ER-mitochondrial interaction [53]. T3 controls the interaction between mitochondria and ER while UCP2 is augmented [47]. In MALFD, we observed the opposite: lower levels of UCP2 secondary to lower T3 augments this interaction, resulting in endoplasmic reticulum stress, which can result in cell apoptosis.

Interestingly, here we can observe an early double harm. T3 regulates the ER-mitochondrial contact interaction, which alters the function of both organelles. However, whether this event can alter mitochondrial activity is not elucidated. Here, we advance in this question showing that the augmented T3 inactivation by D3 can disrupt this interface by altering protein function via disruption of disulfide bonds. In addition, T3 might also affect the transport of fatty acid transport [54], as the MAFLD group showed a significant reduction in their respiratory capacity (Figure 5B). One needs to consider a decrease in mitochondrial content [55]. T3 can stimulate the PGC1-α pathway, a protein linked to mitochondrial biogenesis [56]. In this sense, the reduction in the availability of T3 by augmented D3 activity decreases the hepatic mitochondrial content and the capacity of the remaining mitochondria to uncouple. These alterations negatively impact the β-oxidation of fatty acids in the liver, accelerating the progression of the disease to other stages.

Normal T3 levels enable homeostasis and dehydrogenase activity of the Krebs cycle [47]. Despite the impairment of the respiratory chains, we observed an increase in the activity of GDH and α-KGDH enzymes in MAFLD animals. These enzymes, in addition to their energetic function, are of great importance in the urea cycle for ammonia absorption [57]. Translating these results to clinical observations, we know that hyperammonemia is associated with advanced MAFLD. Now we add in knowledge showing that the reduction in T3 availability contributes to augmenting its toxicity, forming a continuous damage cycle that culminates, in the long term, in hepatic encephalopathy [58,59]. 

Data described here suggest an attempt to increase the deamination system due to the harm generated by excess ammonia already in the early stages of the disease. This might be due to the diminished T3 levels, among other factors. The importance of GDH and α-KGDH in this process was also observed in a hepatic model of GDH-knockout, where hepatic deletion of GDH triggered a systemic increase in ammonia, in response to reduced urea detoxification and increased ammonia production [60]. Moreover, GDH is released by injured hepatocytes, catalyzing a blood reaction that consumes ammonia and α-KGDH to generate glutamate. This process continues until the total consumption of α-KGDH in the blood. In this process, there is a margin of therapeutic opportunity with antioxidants and agents that absorb ammonia that can bring good results in preventing the disease complications [57].

The whole set of these results brings a new perception of the impact of increased inactivation of the T3 hormone in the liver, caused by augmented D3 activity. This new information directly linking D3 to MAFLD may bring new insights into the mechanisms involved in the disease, in addition to opening a new window of opportunity in the treatment of MAFLD.

## 5. Conclusions

Taken together, our findings demonstrate that the deposition of fat in the liver early on MAFLD, leads to inflammation and imbalance in the REDOX state, resulting in increased D3 activity and diminished T3 levels. The lack of T3 leads to mitochondrial dysfunction and altered α-KGDH in the ammonia cycle, which can contribute to the deterioration of the patient’s clinical condition. The ammonia and T3 ratio open a new therapeutic window to correct the increased D3 and complications as hepatic encephalopathy. The set of results suggest a new and very important role of thyroid hormone metabolism in MAFLD.

## Figures and Tables

**Table 1:** Oligonucleotides used.

*Primers*	*Foward*	*Reverse*
*D1*	5′-ATTTGACCAGTTCAAGAGACTCGTAG-3′	5′-GGCGTGAGCTTCTTCAATGTA-3′
*D3*	5′-TTCCAGAGCCAGCACATCCT-3′	5′-ACGTCGCGCTGGTACTTAGTG-3′
*Ucp2*	5′-TCAACTGTACTGAGCTGGTGACCTA-3′	5′-GGAGGTCGTCTGTCATGAGGTT-3′
Ciclofilin A	5′-GTCAACCCCACCGTGTTCTTC-3′	5′-ACTTGCCACCAGTGCCATTATG-3′

**Table 2:** Biochemical data at the end of the experiment.

Variables	Control	MAFLD	*p* Value
**Weight** **(g)**	535.5 (±45.14)	644.2 (±42.15)	<0.01
**Glucose** **(mg/dL)**	278.2 (±56.76)	353.4 (±64.30)	<0.05
**Triglycerides** **(mg/dL)**	79.33 (±14.71)	105.6 (±23.71)	<0.05
**LDL cholesterol** **(mg/dL)**	16.65 (±2.74)	23.41 (±6.63)	<0.05
**HDL cholesterol** **(mg/dL)**	53.23 (±9.77)	31.27 (±6.63)	<0.01
**Total cholesterol** **(mg/dL)**	78.74 (±11.72)	101.8 (±27.54)	<0.05

## Data Availability

The data presented in this study are available on request from the corresponding author. The data are not publicly available due to that some data is still unpublished.
